# Defining the mechanisms of action and mosquito larva midgut response to a yeast-encapsulated orange oil larvicide

**DOI:** 10.1186/s13071-022-05307-6

**Published:** 2022-05-28

**Authors:** Patrick H. Kelly, Alexandra V. Yingling, Anwar Ahmed, Ivy Hurwitz, Marcelo Ramalho-Ortigao

**Affiliations:** 1 Department of Preventive Medicine and Biostatistics, Uniformed Services University, Bethesda, MD USA; 2 Center for Global Health, University of New Mexico Health Sciences Center, Albuquerque, NM USA

**Keywords:** *Aedes aegypti*, YEOO, Essential oil, Larvicide, Gene expression, Mosquito larva, Midgut

## Abstract

**Background:**

Yeast-encapsulated orange oil (YEOO) is a novel, ingestible larvicide that combines the benefits of a low-cost essential oil with yeast, an attractive food source for mosquito larvae. In this work, we investigated the underlying mechanisms of action associated with YEOO ingestion by *Aedes aegypti* larvae.

**Methods:**

*Aedes aegypti* third-stage larvae (L3) were treated with sublethal or lethal concentrations of YEOO. Genes associated with apoptosis, autophagy and innate immune responses were investigated by RT-qPCR in guts and carcasses dissected from treated and control larvae. Differential expression of cytochrome P450 genes in the *CYP6* and *CYP9* families were also investigated. Confocal and transmission electron microscopy were used to assess damage caused by YEOO throughout the larval alimentary canal. TUNEL was used to assess apoptosis via DNA fragmentation.

**Results:**

The apoptosis genes *IAP1* and *IAP2* in larvae displayed opposing effects following exposure to lethal doses of YEOO, with a 26-fold induction of *IAP1* at 8 h post YEOO ingestion. The effector caspase *CASPS8* displayed a 6.7-fold induction in the gut and concomitant 70-fold induction in the carcass at 8 h post YEOO ingestion. The midgut epithelia regenerator, *Vein*, had an 11-fold induction in the gut after 4 h and was repressed 7.6-fold in the carcass at 24 h. Sublethal concentrations (< LC_50_) led to significant differential expression of* CYP6* and* CYP9* genes. Midgut epithelial damage was highlighted by the destruction of microvilli, vacuolization of midgut cells and damage to cell junctions and basal lamina as early as 30 min. Larval type 2 peritrophic matrix structural integrity and porosity remain unchanged.

**Conclusion:**

Our results strongly suggest that the robust larvicidal activity of YEOO is due to a generalized broad-acting mechanism combining epithelial damage and apoptosis, with concomitant expression of multiple innate response genes involved in epithelial regeneration and detoxification. YEOO’s amenability for use as part of an integrated vector management program makes this novel larvicide a practical approach for mosquito larval control in the future.

**Graphical Abstract:**

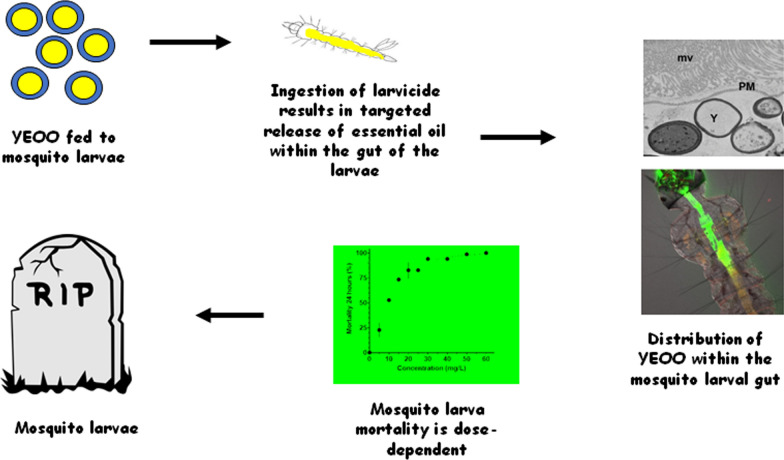

**Supplementary Information:**

The online version contains supplementary material available at 10.1186/s13071-022-05307-6.

## Background

Mosquito-borne diseases continue to represent a major threat to global health and to the economic development of many countries worldwide. Methods to combat these diseases require strategic planning as effective control of mosquito populations are labor intensive, difficult to sustain and fraught with issues of vector resistance. Primary approaches to mosquito abatement includes adulticide spraying with synthetic pyrethroids and organophosphates, larval source management with bacterial larvicides or a combination of both. Control of *Aedes aegypti,* a major vector of dengue, Chikungunya and Zika, has posed a tremendous challenge for vector-control programs. The endophilic behavior of this species in urban areas has allowed for low mosquito populations to sustain disease outbreaks. Initial successes achieved with insecticide control of *Ae. aegypti* have been severely hampered by the emergence of resistance. Recently, methods that apply either transgenic technology via the release of insects carrying a dominant lethal (RIDL) [1, 2] or mosquitoes carrying the endosymbiont Wolbachia to promote cytoplasmic incompatibility of the offspring [3] have been used to reduce mosquito population sizes. However, these methods of control are still in their infancy, are hampered by regulatory barriers, cost and negative public perception and may require several years of investigation to fully assess their perceived potential.

Larvicides have remained an effective means of larval source management. Bacterial larvicides are considered to be more environmentally friendly than chemical insecticides, with fewer or no toxic effects at all on beneficial insects (e.g. bees and butterflies) and other non-target species. As such, this class of larvicides is widely accepted by the general public. Among the most widely used bacterial larvicides, *Bacillus thuringiensis israelensis* (Bti), is highly effective in reducing the risk of mosquito-borne diseases as they can be easily administered in urban and rural standing water. However, Bti has been shown to be unstable after 48 h following application [4]. Furthermore, resistance to *Bacillus sphericus*, another commonly used bio-larvicide, has been reported [5, 6].

Multiple novel approaches for mosquito control are currently being evaluated, including the use of natural essential oils (EOs) [7–11]. EOs are volatile, aromatic oils that have long been used for many purposes, including medicinal, bactericidal, insecticidal, agricultural and pharmaceutical applications. They possess robust mosquito larvicidal traits. Many of the primary components of EOs have been demonstrated to exert their larvicidal effects through at least three different mechanisms: neurotoxicity [12, 13], growth inhibition and interruption of metabolic pathways [14–16]. However, EOs are hydrophobic in the aquatic environment and susceptible to breakdown not only from ultraviolet light but also temperature and oxidation [17], highlighting a need to develop an efficient yet effective delivery system to utilize their potency against mosquito larvae. *Saccharomyces cerevisiae* (Baker’s yeast) can serve as a suitable and effective biodegradable container of various compounds, including medications, fungicides and EOs [18–20]. The encapsulation of EOs renders the yeast cells nonviable, but increases water solubility, bioavailability, long-term viability and stability as a lyophilized material, as well as delivery efficiency as a mosquito larvicide. Further, yeast is readily consumed by mosquito larvae. Specialized intestinal enzymes within the larvae gut can rapidly digest the β-1,3-glucans in the yeast cell wall [21], resulting in the targeted release of EO within the gut. In previous work, our team demonstrated that yeast-encapsulated orange oil (YEOO) is an effective larvicide against multiple larval life stages of *Ae. aegypti* and *Culex quinquefasciatus* [20]. Moreover, we showed that the YEOO LC_50_ and LC_90_ (lethal concentration/dosage leading to 50% and 90% mortality in test organisms) in third-stage larvae (L3) of *Ae. aegypti* after 24 h of exposure to YEOO are 12 and 28 ppm, respectively, which is nearly half of the required dose necessary when using non-encapsulated orange oil [22].

In the present study, we sought to better understand the mechanism of action of our novel larvicide. While innate response gene expression profiles have been extensively investigated in adult mosquitoes [23–26], little is known on the innate responses of mosquito larvae following exposure to larvicides in general. In this article, we describe the changes in innate response gene expression profiles and report our observations on damage inflicted to the midgut epithelial cells in *Ae. aegypti* larvae following YEOO ingestion. The rapid action of our larvicide is hypothesized to result in the activation of apoptotic pathways and epithelial regeneration networks, as well as in the upregulation of detoxification mechanisms linked with the expression of cytochrome P450 (CYP) genes. Our results appear to suggest that ingested orange oil results in substantial midgut epithelial damage that leads to larval death.

## Methods

### Larvicide preparation

Lyophilized yeast-encapsulated orange oil was prepared as previously described [20]. Briefly, *S. cerevisiae* (Red Star fresh baker’s yeast), orange oil (*Citrus sinensis*, California origin; Sigma-Aldrich, St. Louis, MO, USA) and water were combined in a baffled flask at a ratio of 1:5:16 by weight [20] and the flask agitated for 24 h at 40 °C. The resulting mixture was then centrifuged, and the supernatant discarded. The remaining larvicide was washed to remove excess oil and lyophilized prior to storage. Freeze-dried larvicide was reconstituted in water to between 4.4% and 5% oil in solution and diluted appropriately prior to application.

### Larval strains and bioassays

The larvae used in the bioassays were obtained from mosquito colonies maintained at Uniformed Services University of the Health Sciences (Bethesda, MD, US). The bioassays were performed using L3 of *Ae. aegypti* Liverpool strain (AAE-L) and Washington, D.C. strain (AAE-DC); for all other experiments, L3 of AAE-L were used. Larvae were maintained in deionized (DI) water at 28 °C and were fed fish food ad libitum. Larvae bioassays were performed according to the standard WHOPES protocol [27]. Briefly, 25 L3 larvae were placed into cups containing 100 ml of DI water. After a 30 min acclimation, YEOO larvicide was added to each cup to reach concentrations ranging from 2.5 to 60 mg/l. The cups (L3) were assessed after 24 h to determine YEOO toxicity.

For the gene expression and morphology studies, L3 were treated with 5, 10 and 30 mg/l YEOO, respectively. These concentrations correspond to the LC_20_, LC_50_ and LC_90_, respectively, of YEOO against these insects based on our previous studies [20]. The effects of YEOO on AAE-L L3 larvae in terms of swimming behavior and lethality are shown in Additional file 1: Video S1; Additional file 2: Video S2; Additional file 3: Video S3.

### Gene expression analyses

Genes associated with apoptosis (*IAP1* and *IAP2*), autophagy (*ATG1*, *ATG6*, and *ATG8*) and innate immune responses (*Vein*, *Pirk*, *Serpin-1*, *Serpin-2*, *IMP2*, *CASPS7* and *CASPS8*) were assessed in guts and carcasses dissected from L3 following 1, 4, 8, and 24 h exposure to YEOO either at a sublethal dose (10 mg/l, LC_50_) or at a lethal dose (30 mg/l, LC_90_). Gut samples comprised dissected foregut and midgut, and carcass samples included the gut samples plus the hindgut, Malpighian tubules and all remaining larvae-matched body parts (i.e., head, thorax and abdomen). Guts or carcasses from three individual larvae were pooled at each time point; each experiment was performed twice. Collected samples were placed into 30 µl of RNAlater (Thermo Scientific, Waltham, MA, USA) and frozen at − 80 °C until RNA extraction.

To assesses the possibility of resistance to YEOO, members of the CYP superfamily, represented by the *CYP6* family (*CYP6M11*, *CYP6N12*, and *CYP6Z8*) and *CYP9* family (*CYP9J10* and *CYP9M9*), were investigated in L3 following YEOO treatment with sublethal doses (5 or 10 mg/l) of YEOO for 4 h. Whole larvae were used in these experiments for RNA isolation as CYPs are known to be expressed in the midgut and elsewhere in mosquitoes, including the fat bodies and Malpighian tubules [28, 29]. In these experiments, three replicate experiments were performed, and up to nine individual larvae were collected in each experiment.

### RNA extraction and complementary DNA synthesis

Total RNA was isolated using the RNeasy tissue kit (Qiagen, Hilden, Germany) followed by DNase treatment using TURBO DNA-free kit (Invitrogen, Thermo Fisher Scientific, Waltham, MA, USA). Extracted RNA was quantified on a NanoDrop (Thermo Fisher Scientific) and assessed for integrity by gel electrophoresis. RNA samples were stored at − 80 °C until use. Complementary DNA (cDNA) synthesis was performed using the Superscript III kit (Invitrogen, Thermo Fisher Scientific) following the manufacturer’s protocols with oligo dT_12-20_ primers and 200 ng of each RNA.

### Real-time quantitative PCR

cDNAs were amplified using the primer pairs listed in Additional file 4: Table S1. Real-time quantitative PCR (RT-qPCR) was performed on an Applied Biosystems™ 7500 Real-Time PCR System (ABI7500 FAST; Applied Biosystems, Thermo Fisher Scientific, Waltham, MA, USA) using the PowerUp SYBR Green Supermix (Applied Biosystems). All reactions were initiated with a hot start of 50 °C for 2 min and 95 °C for 2 min, followed by 40 cycles of 60 °C for 5 s and 95 °C for 15 s. All RT-qPCR assays were performed in triplicate. Relative fold changes were assessed using the 2^−∆∆CT^ method [30] and calibrated against the expression of the housekeeping gene *Actin6* (Additional file 4: Table S1) in control L3 that were fed a similar concentration of inactivated yeast. Inactivated yeast was prepared similarly to YEOO, but in the absence of any EOs.

### Confocal microscopy

Confocal microscopy was used to assess the distribution of YEOO throughout the alimentary canal of larvae. Whole guts from L3 were dissected after exposure to either 30 mg/l YEOO or a similar concentration of inactivated yeast (control) for 4 h, and fixed for 15–30 min at room temperature with Zamboni’s fixative [31].

To assess whether ingestion of YEOO affected the permeability of the type 2 peritrophic matrix (PM2), L3 were treated with either 30 mg/l YEOO or inactivated yeast in water that was supplemented with 0.5 mg/ml FITC-dextran (molecular weight [MW]: 150, 500 or 2000 kDa; Cell Signaling Technology, Danvers, MA, USA). After 4 h of treatment, alimentary canals were dissected from larvae of both treatment groups and fixed in Zamboni’s fixative [31] as described above. Following three washes in phosphate buffered saline (PBS), the tissues were stained for 5 min with 10 µg/ml of DAPI (Invitrogen™, Thermo Fisher Scientific, Waltham, MA, USA). Samples were mounted onto charged slides after more washes in PBS and subsequently imaged on a ZEISS 710 Two Photon confocal microscope (Carl Zeiss AG, Hoberkochen, Germany). The permeability of larval PM2 was qualitatively assessed by presence/absence of fluorescein signal in the caeca of dissected guts/larvae [32].

### Terminal deoxynucleotidyl transferase dUTP nick end labeling analysis

Terminal deoxynucleotidyl transferase dUTP nick end labeling (TUNEL) was used to assess apoptosis via DNA fragmentation. L3 were exposed to either YEOO (10 mg/l) or a similar concentration of inactivated yeast for 24 h. Guts were dissected and fixed in Zamboni as described above. Tissues were washed three times (5 min each) in PBS, twice (2 min each) in PBS containing 0.3% Triton X-100 (PBST), followed by TUNEL analysis (Roche, Basel, Switzerland) according to manufacturer’s protocol. Samples were counterstained with 10 µg/ml of DAPI (Invitrogen), mounted onto slides in Vectashield™ (Vector Laboratories, Burlingame, CA, USA), and subsequently imaged on a ZEISS 710 Two Photon confocal microscope (Carl Zeiss AG).

### Transmission electron microscopy

To determine cellular damage caused by YEOO ingestion, L3 were exposed to 30 mg/l of either YEOO or inactivated yeast (control). Following 4 h exposure, alimentary canals were dissected from both treatment groups. Tissues were fixed overnight at room temperature in freshly prepared 2% formaldehyde and 2% electron microscopy (EM) grade glutaraldehyde (Electron Microscopy Sciences, Hatfield, PA, USA) in 0.1 M cacodylate buffer, pH 7.2. Fixed tissues were washed three times, for 10 min each, in cacodylate buffer (without aldehydes) prior to incubation for 1 h in 2% OsO4. After more washes in 0.1 M cacodylate buffer, tissues were dehydrated in a graduated series of ethanol (10 min each in 30%, 50%, 70%, and 95% ethanol followed by 2 × 10 min in 100% ethanol), infiltrated in a graduated series of Spurr’s epoxy resin (Electron Microscopy Sciences) and then polymerized at 70 °C for 11 h. Polymerized blocks were sectioned in a Leica UC6 ultramicrotome (Leica Microsystems GmbH, Wetzlar, Germany). Longitudinal and transverse thin sections were collected on 3-mm copper grids. Grids were post-stained in a Leica EM AC20 grid stainer (Leica Microsystems GmbH) and then examined on a JEOL JEM-1011 transmission electron microscope (JEOL USA, Peabody, MA, USA). Images were collected on an Advanced Microscopy Techniques digital camera (AMT Corp., Woburn, MA, USA).

Transmission electron microscopy (TEM) was also utilized to assess the structural integrity of YEOO. Similar methodologies were utilized prepare YEOO samples for examination by TEM.

### Statistical analysis

Larvae bioassay data to determine the LC_50_ and LC_90_ YEOO effective concentrations were determine with Probit regression analyses [33]. Two-way analysis of variance (ANOVA), with Dunnett’s multiple comparisons tests was used to determine variation in gene expression profiles in the guts or carcasses of either YEOO- or control-treated larvae over time. In the whole larvae CYP gene expression analyses, data were subjected to one-way ANOVA and Tukey’s post-hoc tests. Statistical analyses were carried out using the SAS version 9.4 (SAS Institute, Cary, NC, USA) or GraphPad Prism 8 (GraphPad Software, San Diego, CA, USA) software packages.

## Results

### Bioassays

**Fig. 1:**
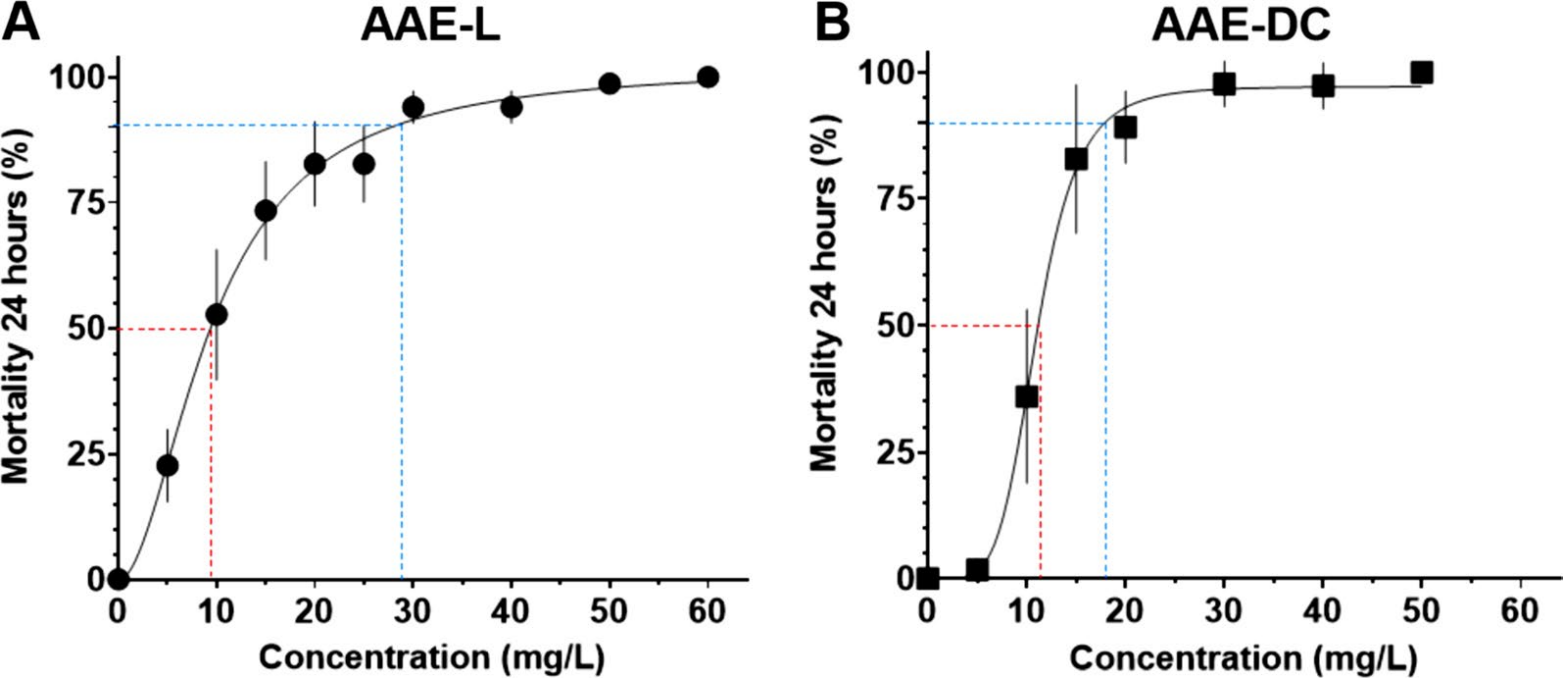
YEOO in larval killing bioassay. Each larval killing bioassay was performed with 25 L3, which were placed in a plastic cup containing 100 ml deionized water and the appropriate concentration of YEOO larvicide for 24 h.** a** *Aedes aegypti* strain Liverpool (AAE-L),** b** *Ae. aegypti* strain Washington DC (AAE*-*DC)*.* Mortality was assessed using the logistic procedure in GraphPad Prism 8 software. Dose–response curves were plotted with nonlinear regression best fit with the means and SEM of each concentration tested. LC_50_ (red dotted line) and LC_90_ (blue dotted line) are shown. A minimum of two and up to eight replicates (N) were performed for each concentration tested. Abbreviations: LC_50_, LC_90_, lethal concentration/dosage leading to 50% and 90% mortality; L3, third-stage larva; SEM, standard error of the mean; YEOO, yeast-encapsulated orange oil

YEOO was effective against L3 of both *Ae. aegypti* strains (AAE-L and AAE-DC). For strain AAE-DC, the LC_50_ and LC_90_ were calculated to be 11.1 and 17.9 mg/l, respectively (Fig. 1; Additional file 4: Table S1). For the AAE-L strain, the LC_50_ and LC_90_ were in agreement with the results reported in our previous study [20], i.e. 9.4 and 27.8 mg/l, respectively. The sublethal dose of LC_20_ was calculated to be 5 mg/l from these plots. YEOO was also found to be effective against *Anopheles gambiae* (strain G3), with an LC_50_ and LC_90_ of 10.3 and 28.1 mg/l, respectively (Additional file 5: Fig. S1).

### Gene expression profiles

Expression of genes associated with apoptosis, autophagy and innate immune responses were examined in the guts and carcasses of L3 at various time points following exposure to two concentrations of YEOO (10 mg/l [LC_50_] or 30 mg/l [LC_90_]). Samples were collected only from motile larvae; no moribund or dead larvae were included for gene expression analyses. Gene expression profiles from the comparisons of guts versus carcasses and across the time points assessed were found to be more robust with the LC_90_ treatment. For the LC_50_ treatment, the statistical analyses revealed differences to be non-significant, with standard error of the mean values similar to those of the observed fold-changes (data not shown).

**Fig. 2:**
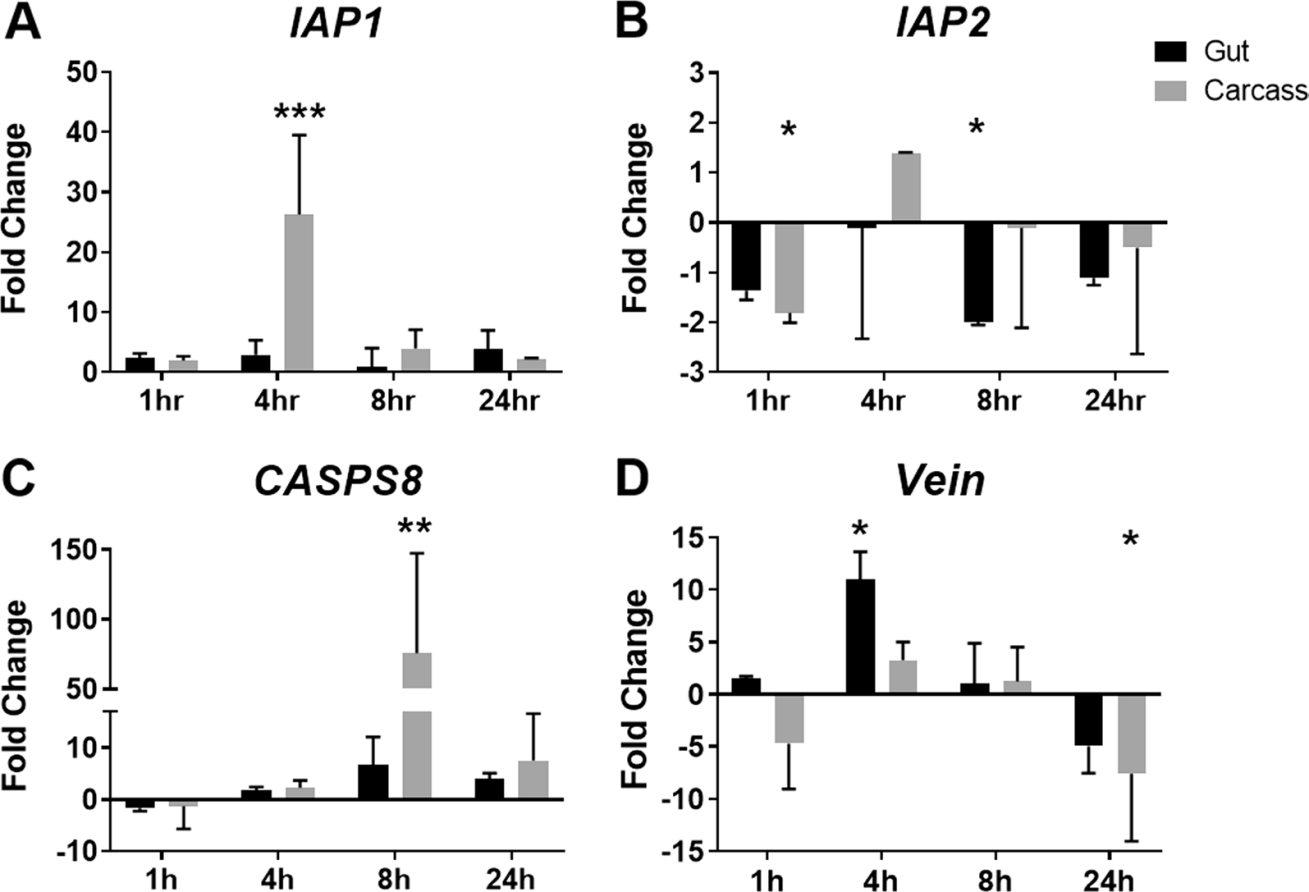
mRNA expression profiles in L3 after exposure to YEOO LC_90_ (30 mg/l). Spatial expression of innate response genes in the mosquito larvae. Black bars represent the dissected gut; gray bars represent the carcass. Guts or carcasses from 3 individual larvae were pooled at each time point. These experiments were performed twice. Results were analyzed using two-way ANOVA with Dunnett’s multiple comparisons post-test. Statistical differences at each time point are shown as *P < 0.05, **P < 0.01, ***P < 0.001. Abbreviations: ANOVA, Analysis of variance; *IAP1*, *IAP2*, genes associated with apoptosis; *CASPS8*, *Vein*, genes associated with innate immune responses; mRNA, messenger RNA

Larvae exposed to YEOO larvicide LC_90_ displayed opposing effects for *IAP1* and *IAP2*. For *IAP1*, a 26-fold induction in the carcass at 8 h (Fig. 2a; *P* = 0.0001) was observed, whereas for *IAP2* we detected a significant reduction in the carcass at 1 h (Fig. 2b; *P* = 0.0389) and in the gut at 8 h (Fig. 2b; *P* = 0.0298). For the effector caspase *CASPS8*, a 6.7-fold induction in the gut and concomitant 70-fold induction in the carcass were observed at 8 h post-YEOO ingestion (Fig. 2c; *P* = 0.0071). For the midgut epithelia regenerator *Vein*, a significant increase, by 11-fold, was observed in the gut after 4 h (Fig. 2d; *P* = 0.0094), followed by repression, by 7.6-fold, in the carcass tissues by 24 h (Fig. 2d; *P* = 0.0227).

Other transcripts examined, including *Pirk*, a negative regulator of the IMD innate immunity pathway; *Serpin-1*, *Serpin-2* and *IMP-2*, genes associated with melanization cascade; *ATG6,* an autophagy-associated gene; and *CASPS7*, an effector caspase, did not reveal any suggestion of differential expression following YEOO exposure (data not shown).

**Fig. 3:**
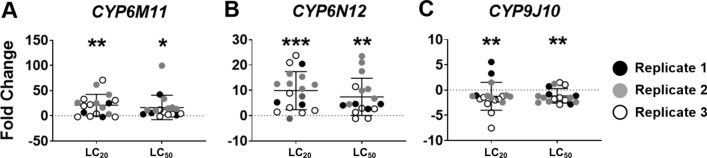
Cytochrome P450 mRNA expression in L3 after exposure to either YEOO LC_20_ or LC_50_ for 4 h. Expression of *CYP6M11, CYP6N12* and *CYP9J10* were assessed in whole larvae after a 4-h exposure to the two concentrations of YEOO. Three replicate experiments were performed. Up to nine individual larvae were collected in each experiment. Results were analyzed using one-way ANOVA with Tukey’s post-hoc analyses. Statistical differences are shown as *P < 0.05, **P < 0.01, ***P < 0.001. Abbreviation: CYP, Cytochrome P450; LC_20_ lethal concentration causing 20% mortality

We next assessed whether expression of several drug-detoxifying enzymes were affected by exposure to sublethal concentrations (LC_20_ or LC_50_) of YEOO. Whereas the genes encoding CYP6M11 and CYP6N12 showed upregulation following a 4-h exposure to either concentration of YEOO, the gene encoding CYP9J10 displayed a significant downregulation (Fig. 3). CYP6M11 was upregulated by 21-fold (*P* = 0.0053) and 16.5-fold (*P* = 0.03), while CYP6N12 was upregulated by 9.9-fold (*P* = 0.0002) and 7.4-fold (*P* = 0.007) following LC_20_ and LC_50_ treatments, respectively. CYP9J10 was downregulated at both LC_20_ (*P* = 0.001) and LC_50_ (*P* = 0.002). Two additional CYP genes, those encoding CYP6Z8 and CYP9M9, displayed no significant changes at either sublethal concentration (data not shown).

### Analysis of YEOO integrity

**Fig. 4:**
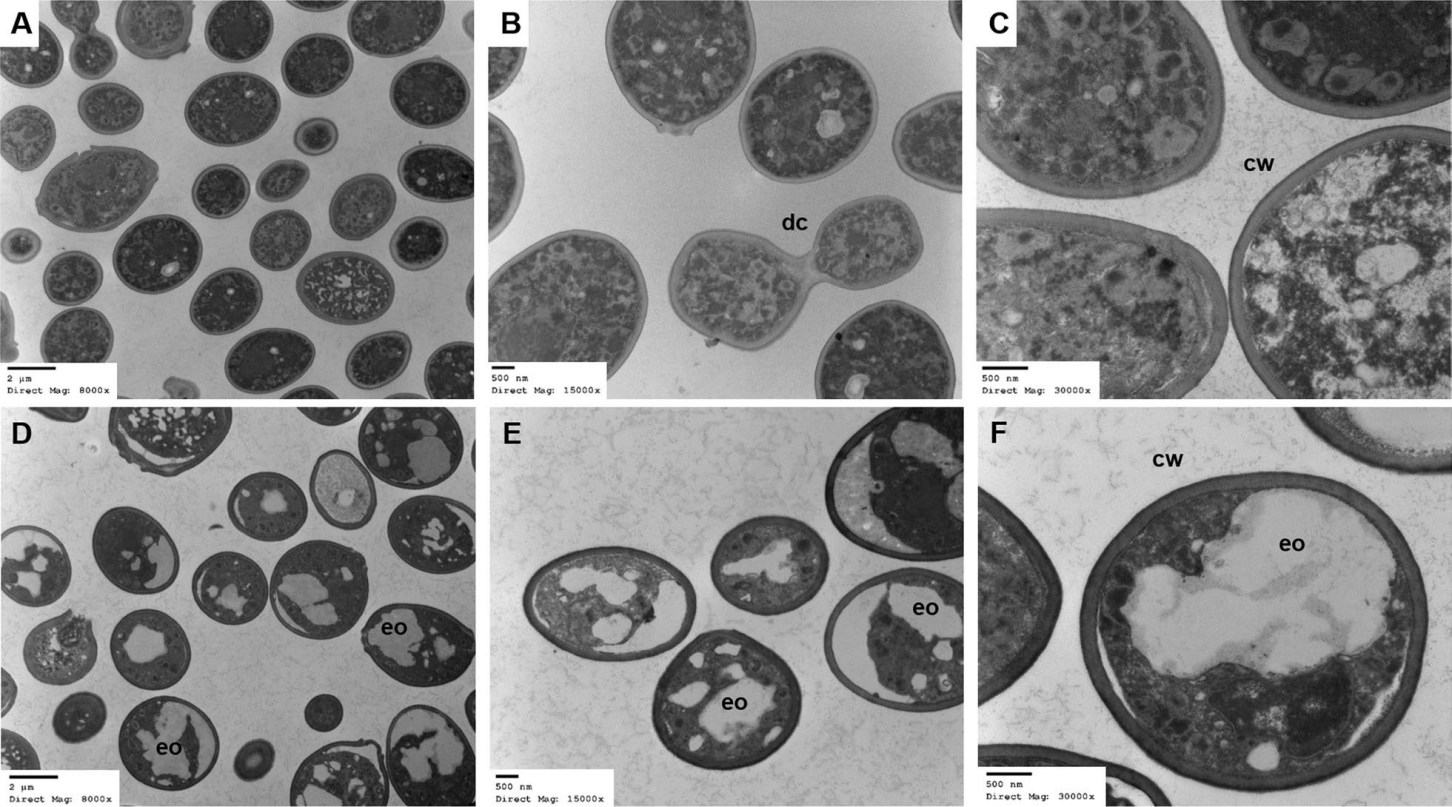
Assessment of yeast cell integrity by TEM. Lyophilized inactivated yeast (control) or YEOO were rehydrated in water at 4.4% for 30 min before fixation and subsequent imaging. **a**–**c** Inactivated yeast (control),** d**-**f** YEOO. Control yeast shows dividing cells (*dc*), not present in YEOO. Panels depicting YEOO show the widespread but uneven loading of the orange oil (*eo*). The cell wall (*cw*) from the control and YEOO are intact and unaffected by essential oil. Magnification: ×8000 (**a**, **d**); ×15,000 (**b**, **e**); ×30,000 (**c**, **f**). Abbreviations: TEM, Transmission electron microscopy

Transmission electron microscopy was utilized to verify the structural integrity of YEOO. Compared to inactivated yeast controls (Fig. 4a–c), nearly all *S. cerevisiae* cells were loaded with EOs (Fig. 4d–f). As expected, no cell division was observed in YEOO as the encapsulation process effectively killed the yeast cells. Bud scars, which were visible in this population, likely occurred before EO loading.

### Cellular and physiological damage in midgut after YEOO exposure

**Fig. 5:**
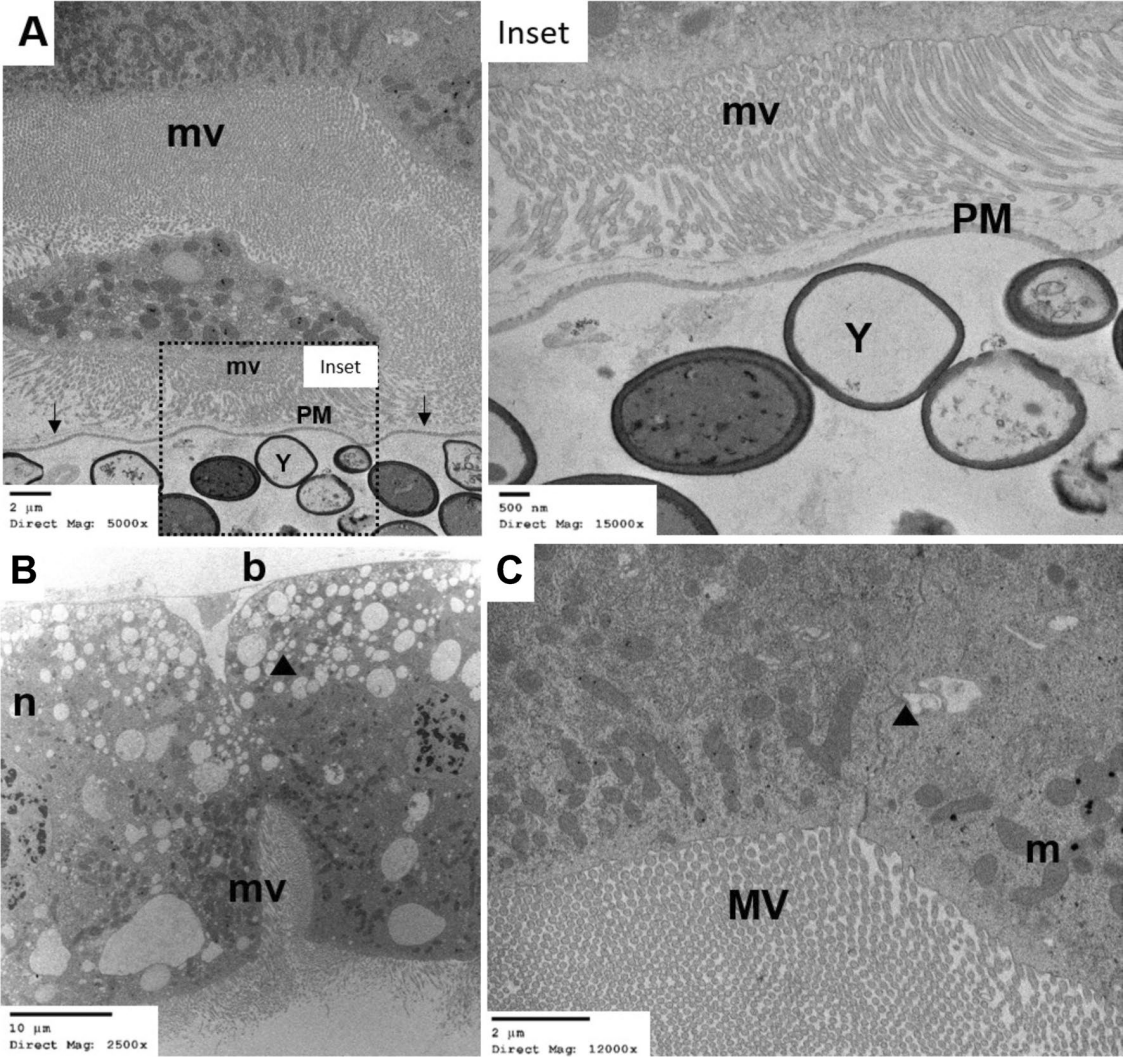
TEM of larval midgut fed inactivated yeast cells. **a** Normal appearance of gut of L3 fed on inactivated yeast cells (*Y*) sequestered in the endoperitrophic space. The PM2 (arrows) and extensive microvilli (*mv*) are visible. Inset shows mv and the PM2 in greater detail. **b** Midgut epithelial cell showing cell junctions (arrowhead) between adjacent cells, mv, and basal lamina (*b*). The nucleus (*n*) and integral nuclear membrane are also visible. **c** Greater detail of midgut epithelial cells with clearly distinguishable cell junction (arrowhead), mitochondria (*m*) and microvilli (*MV*). Abbreviations: PM, Type 2 peritrophic matrix

**Fig. 6:**
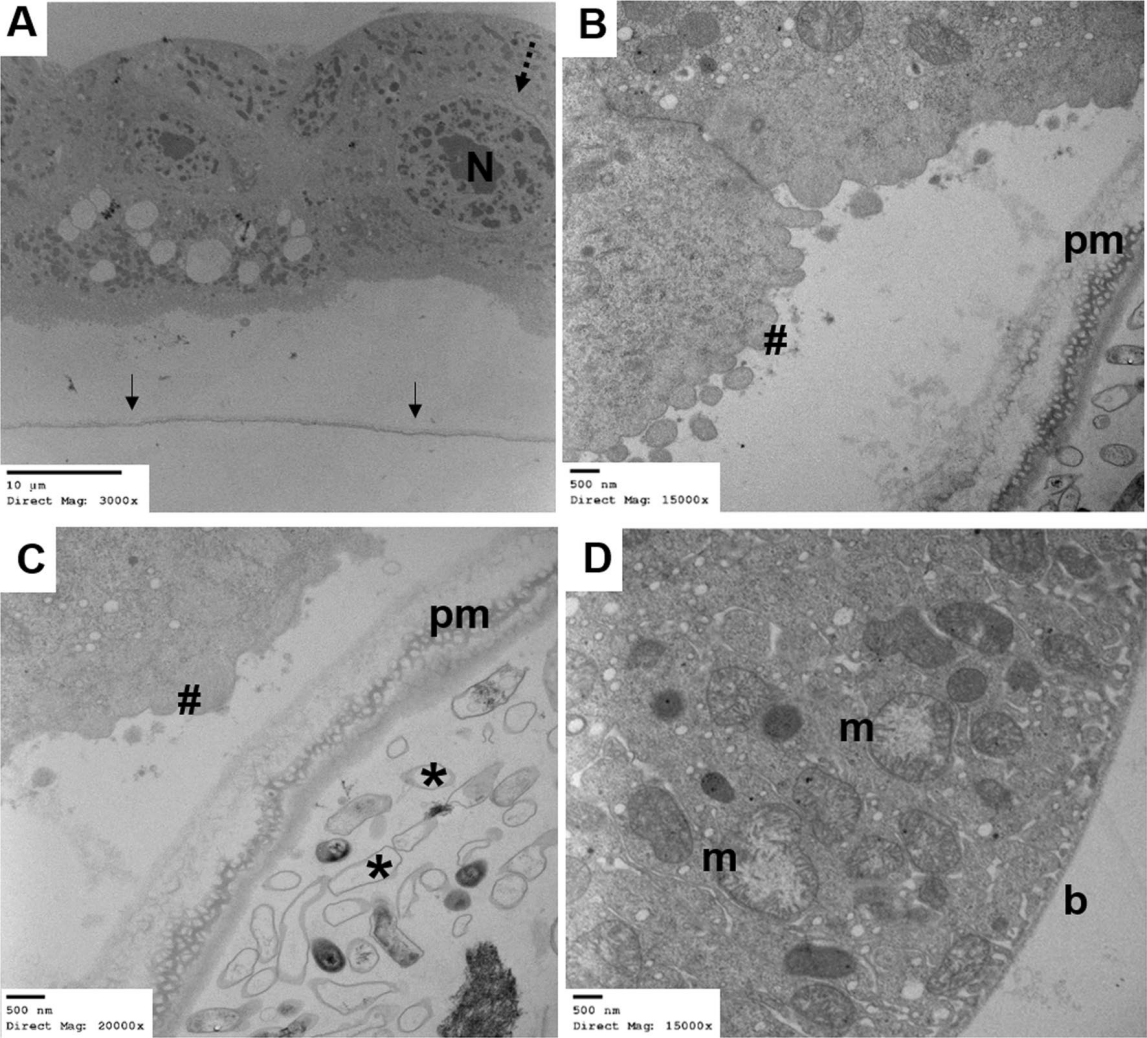
TEM of larval midgut after 30 min of YEOO LC_90_ exposure (30 min after ingestion of YEOO). **a** No detectable microvilli and increased blebbing in the nucleus (*N*) and incipient fragmentation of the nuclear membrane (dashed arrow), with the PM2 still visible (arrows). **b** Greater magnification of apical cell surface with loss of microvilli (#) and blebbing of cell membrane with the PM2 (*pm*) still intact. **c** Detailed view of the apical epithelium (#) with significant destruction of the microvilli network. No YEOO are visible within the peritrophic space (*) surrounded by the PM2 (*pm*. **d** Slightly increased vacuolization is seen near the basal lamina (*b*) of midgut epithelial cells with still uniform mitochondria (*m*)

**Fig. 7:**
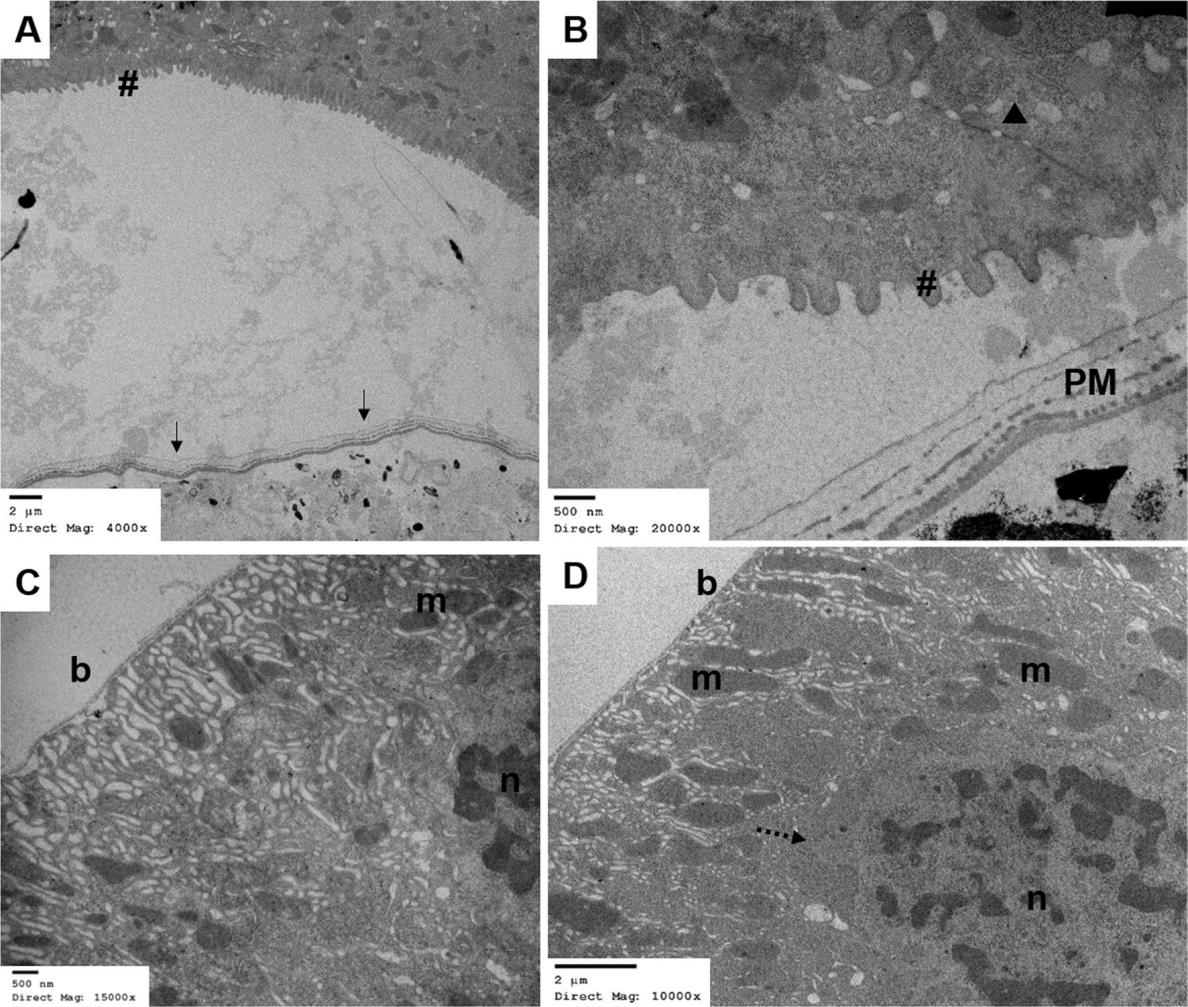
TEM of larval midgut after 4 h YEOO LC_90_ exposure (4 h after ingestion of YEOO). **a** Complete loss in microvilli (#), increased vacuolization within epithelia cytosol and no YEOO cells visible within peritrophic space. **b** Greater detail of the apical portion of epithelial midgut cells showing complete loss of microvilli and blebbing (#), uncharacteristic cell junction (arrowhead), yet intact PM2 (*PM*). **d**, **e** Basal lamina (*b*) of epithelial cell displaying increased vacuolization, blebbing of the nucleus (*n*), fragmentation of the nuclear membrane (dashed arrow) and abnormal or irregularly shaped mitochondria (*m*)

**Fig. 8:**
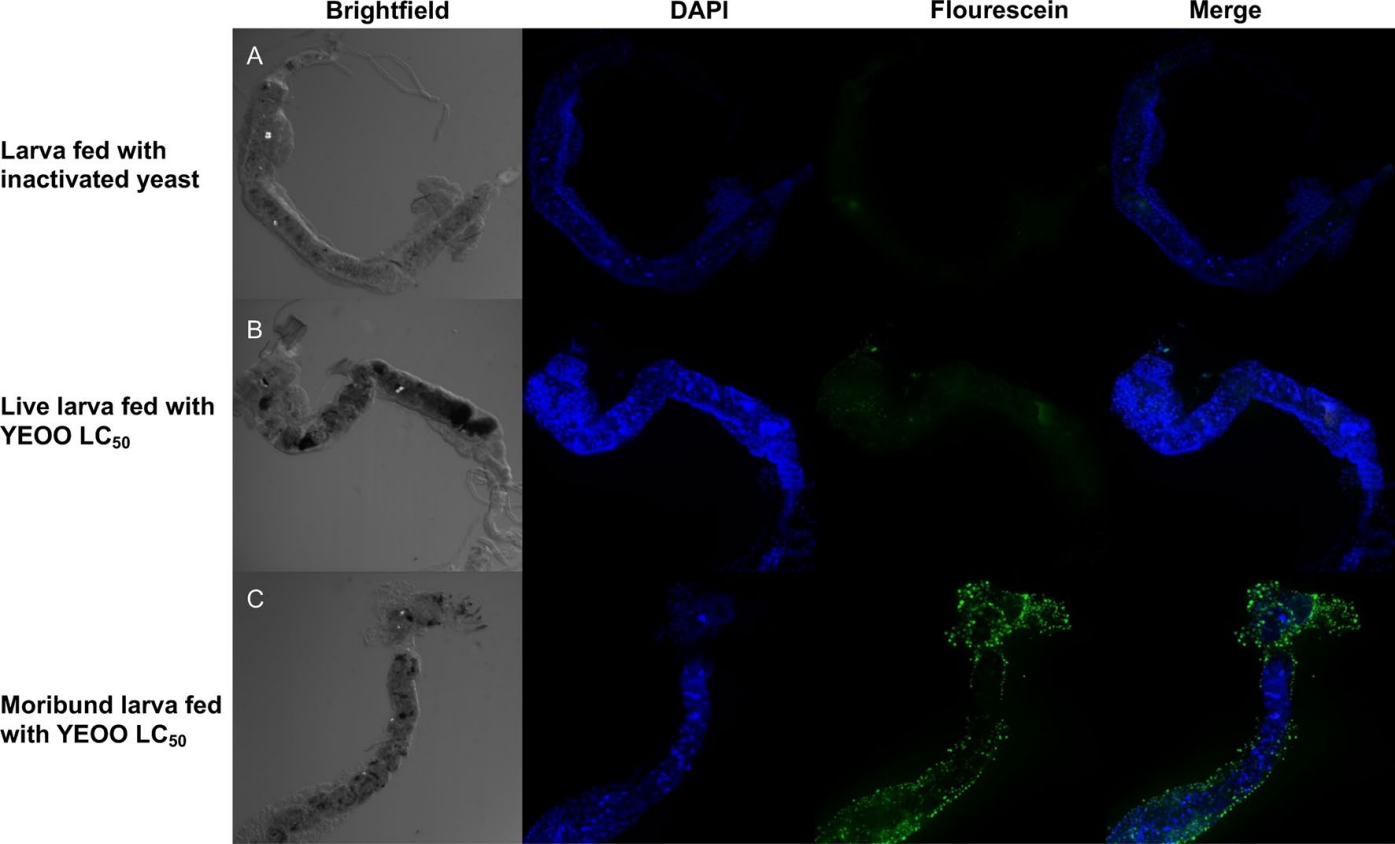
TUNEL assay of larval midgut after sublethal exposure to YEOO (LC_50_) for 24 h. **a** Dissected midgut from larvae fed on inactivated yeast, **b**, **c** dissected midguts from either live (**b**) or moribund (**c**) larvae fed YEOO LC_50_. Increased DNA fragmentation is seen in the midgut of moribund larvae. Abbreviations: TUNEL, Terminal deoxynucleotidyl transferase dUTP nick end labeling

Figure 5 shows ingested inactivated yeast within the larvae midgut surrounded by the PM2 (Fig. 5a and Inset). No ultra-cellular damage was observed at the level of the microvilli (mv), cell junctions (arrowhead), mitochondria (m), nuclei (n) or fat vacuoles throughout the midgut cells (Fig. 5). In contrast, there was substantial damage to midgut epithelial cells within 30 min of exposure to YEOO LC_90_ (Fig. 6), followed by significant damage systemically throughout the midgut epithelia after 4 h (Fig. 7). Microvilli were severely damaged/shortened at the 30-min exposure (Fig. 6a–c) or completely destroyed with the 4-h exposure (Fig. 7a, b). An increase in cellular vacuolization was also noted, particularly along the basal lamina (b in Figs. 7d, 8c, d), as well as alterations in the mitochondrial shape and cristae (m in Figs. 7d, 8c and d). Interestingly, unlike larvae treated with inactivated yeast, no intact YEOO cells were visible within the PM2 of treated larvae regardless of treatment duration (30 min or 4 h).

The hypothesis that the breakdown of YEOO in larval guts might be due to pH was then tested, as no difference in the levels of digestive proteases had been observed (Fernando Genta, personal communication). As the mosquito larval gut pH ranges from 10 to 7.5 from the anterior to posterior regions [34], YEOO larvicide was incubated with in HEPES at incremental increases of pH from pH 7 to pH 10 for 30 min. No changes in the YEOO stability were observed (data not shown).

Lastly, both live and moribund larvae were subjected to TUNEL analysis after 24 h of sublethal exposure to YEOO LC_50_. Guts of moribund larvae exhibited increased DNA fragmentation when compared to live larvae (Fig. 8).

**Fig. 9:**
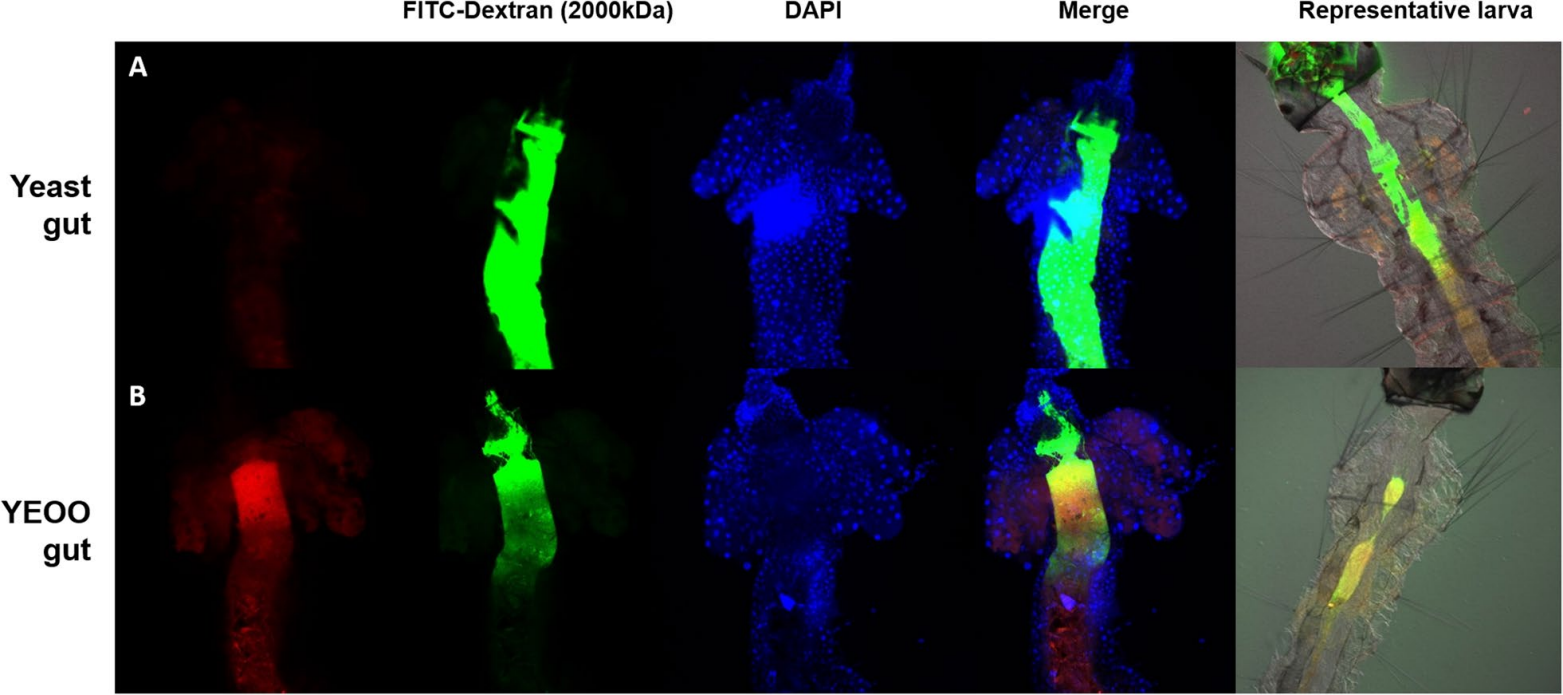
YEOO and PM2 permeability. Confocal microscopy of dissected midguts from larvae fed either on inactivated yeast (**a**) or YEOO LC_90_ (**b**) for 4 h in conjunction with FITC-labeled dextran (MW: 2000 kDa). No FITC-dextran label (MW: 2000 kDa) was observed in the caeca of either control or YEOO-treated larvae, suggestive of no change in PM2 permeability

Although the structural integrity of the PM2 did not appear to be affected by YEOO at LC_50_ for up to 4 h, we assessed if the PM2 permeability or porosity were altered. Under normal physiological conditions, the PM2 in *Ae. aegypti* larvae is not permeable to 2000-kDa FITC-labeled dextran particles [32]. L3 were fed either with inactivated yeast or LC_90_ YEOO together with 2000-kDa FITC-labeled dextran for 4 h. FITC-related fluorescence was not detected in the caeca of either treatment (Fig. 9). These results suggest that YEOO does not affect the permeability of the *A. aegypti* larval PM2. Although attempts were made to test the permeability of the PM2 after YEOO treatment, the simultaneous feeding described above was our only option as the larvae refused to ingest the FITC-labeled dextran particles after prior exposure to YEOO.

## Discussion

In the present study we demonstrate the effects of YEOO, a novel ingestible mosquito larvicide, on the physiology and innate response gene expression profiles in *Ae. aegypti* L3. Ingestion of the YEOO resulted in cytotoxic events in larval midgut epithelial cells as well as clear upregulation of at least one apoptosis-related gene, *IAP1*. Unlike traditional single-target insecticides, YEOO displays multi-modal action affecting multiple cellular pathways. Thus, we predict that YEOO activity is independent of specific midgut receptors and that its killing mechanisms are based on its toxicity to midgut cells. Multi-target larvicides, such as Bti, or multi-modal larvicides, like YEOO, are thought to be less likely to incur resistance [11, 18, 19]. To date, resistance to EOs has not been reported.

EOs and their derivatives have been investigated in vitro against neglected tropical parasites and arboviruses for decades [35]. They have been utilized as contact irritants against adult and larval stages of various insect pests [36]. Further, they have been shown to cause testicular apoptosis and morphological damage to the fat body and midgut epithelium of *Spodoptera frugiperda* (lepidopteran) larvae [37, 38]. Other natural compounds, such as squamocin, an extract from the Amazonian plant *Derris urucu*, have been tested against *Ae. aegypti* larvae on which both cytotoxic and gene expression profiles effects were assessed [39, 40].

Encapsulated EOs offer a significant advantage over free EOs. Moreover, whereas the hydrophobicity of free EOs makes them unlikely candidates for commercial use. The encapsulation approach using *S. cerevisiae* provides an optimal delivery mechanism that facilitates dissemination throughout aquatic environments. When sequestered into yeast cells, EOs are effectively protected against photolytic degradation, allowing for long-term stability [20]. In the present study, the integrity of the reconstituted YEOO was confirmed via TEM analysis. Finally, upon ingestion by the mosquito larvae, the yeast cell wall is digested by the various β-glucanases [21] present within the midgut of the larvae, allowing for targeted released of the EO payload.

Although our results on gene expression profiles only provide indirect evidence, the increased expression of the midgut epithelial cell regenerator *Vein* at 4 h post YEOO exposure, with concomitant upregulation of *CASPS8* at 8 h post YEOO exposure, is suggestive of an innate response to reconstitute the midgut epithelia damaged by the larvicide. This is also hypothesized to be a last-ditch effort to clear dead and dying cells from the YEOO non-specific broad-acting mode of action. Moreover, the modulation observed in both *IAP1* and *IAP2* is potentially associated with their regulatory roles regarding initiator or effector caspases [41]. Accordingly, *IAP1* has been shown to interact and regulate the expression of the initiator caspase *Dredd* and the effector caspases *CASPS7* and *CASPS8* [42, 43]. These effector caspases carry out proteolysis and disintegration of proteins during cell death [41–43]. With regards to* IAP2*, studies in *Drosophila melanogaster* demonstrated its role during expression of apoptotic-associated genes and the negative regulation of the IMD innate immunity) pathway [44] in a fashion similar to the negative regulator *Pirk* [45].

The cytotoxic effects observed following YEOO ingestion were broad, with drastic alteration in cell morphology at both the cellular and subcellular levels in the insect midgut. While YEOO appeared to have produced no specific alterations of the larval PM2, there was substantial damage to midgut epithelia, including loss of microvilli, disordered cellular junctions and increased cytoplasmic vacuolization in laminar bodies. In the cytosol, increased vacuolization and abnormal morphological alterations of the mitochondria and cristae also were observed.

In general, apoptotic signaling is linked with mitochondrial release of cytochrome* c* and other cofactors to activate effector caspases [46]. The presence of circular-shaped mitochondria observed in our TEM images of midgut cells following YEOO exposure is likely due to the cellular cytotoxicity affecting mitochondrial proteins that coordinate cytochrome* c* release and promote the apoptosis expression cascade suggested by the differential expression of *IAP* and *CASP*, and supportive of apoptosis-mediated killing.

However, it has been previously reported that expression of *ATG1, ATG6* and *ATG8* is correlated with the expression of *V-ATPase* following exposure of *Ae. aegypti* larvae to the plant-derived fatty acid compound squamocin [39]. Following YEOO ingestion, the expressions of *ATG1* or *ATG6,* as well as of *V-ATPase* (data not shown), were not significantly different when compared to those of larvae fed on inactivated yeast. Further, in *D. melanogaster*, autophagic cell death in the midgut is accompanied by markers of apoptosis, such as DNA fragmentation [47]. Similarly, the typical pattern of DNA degradation was observed in our TUNEL analysis in moribund larvae. Although the relationship between the autophagic and apoptotic pathways is not yet clear, increased levels of cytoplasmic vacuolization and cell death were observed within 30 min following the ingestion of YEOO by mosquito larvae, as depicted in our TEM images. Despite a lack of direct evidence, the presence of an autophagic mechanism inducible by YEOO and acting in concert with apoptosis, possibly associated with tolerance mechanisms against the non-specific toxic effects observed, has not yet been discarded.

Following ingestion, YEOO is likely digested by proteases secreted within the larval midgut [21]. We excluded the possibility that the breakdown of the yeast cell wall might have been caused by pH variations within the larval gut by testing YEOO in solutions with increasing pH. No changes in YEOO stability were detected in solutions with the pH ranging from 7 to 10. It remains to be determined if YEOO breakdown is due to a continued action of digestive proteases or whether it involves a combination of factors that create a domino effect resulting in cell wall breakdown and the release of the orange oil.

Insecticide resistance poses a serious threat to the control of mosquito-borne diseases. Traditional vector management programs either increase insecticide applications to kill resistant populations or must switch to another pesticide to achieve control. These approaches have not generated any significant advantages for mosquito control but have instead contributed to the increase of resistance in mosquito vectors. Continuous exposure to organophosphates and pyrethroids [48–50] have resulted in the overproduction of CYP enzymes [48, 51, 52] that are necessary for detoxification of the various pesticides. Distinct mosquito species or strains possess unique detoxification or innate mechanisms to cope with the various pesticide classes. In response to YEOO, differential expression was observed between the genes coding for the CYP6 and CYP9 families, suggesting that these detoxifying enzymes are distinctively regulated according to chemical exposure or challenge. However, the results observed with the CYP profiles, in our view, are not directly associated with YEOO resistance. The broad mode of action demonstrated by YEOO likely limits the possibility of emergence of resistance. We believe that for YEOO resistance to develop, a midgut remodeling process would likely be involved.

Larviciding approaches, such as Bti, which is effective against *Ae. aegypti* larvae, are often still out of reach for many affected communities due to frequent need for reapplications and elevated cost. Thus, gaps exist both in the availability of safe, stable, cost- effective and efficacious alternatives in mosquito control approaches. YEOO mosquito larvicide is easy to produce and is stable after long-term storage [20]. Moreover, YEOO is affordable as it primarily relies on local resources (EOs). It is not yet known which effects, if any, YEOO has on *Ae. aegypti* larval development into adults, including effects on the physiology, fecundity and fertility of the adult mosquito, especially after sublethal YEOO exposure (i.e. LC_20_ concentrations). Experiments focused on understanding these effects in larvae in relation to hormesis [53] are subjects of on-going studies.

## Conclusion

YEOO is highly effective against *Ae. aegypti* larvae through mechanisms involving acute midgut cell damage and apoptotic pathways leading to larval death. Because of its broad mode of action, resistance or tolerance against YEOO is unlikely to develop. YEOO is currently being tested against non-target organisms. Once the range of organisms affected by YEOO is properly identified and its status as an environmentally friendly larvicide ascertained, this class of larvicides shall provide a safe and effective mechanism for mosquito population control.

## Supplementary Information

**Additional file 1: Video S1.** 1 h after LC90 YEOO—AAE-L larval swimming behavior and lethality.

**Additional file 2: Video S2.** 4 h after LC90 YEOO—AAE-L larval swimming behavior and lethality.

**Additional file 3: Video S3.** 24 h after LC90 YEOO AAE-L larval swimming behavior and lethality.

**Additional file 4: Table S1**. Primer sequences used for amplification of target genes.

**Additional file 5: Figure S1.** Bioassay of YEOO against *Anopheles gambiae* (G3), showing LC_50_ and LC_90_ of 10.3 mg/l and 28.1 mg/l respectively.

## Data Availability

Data and material used in the studies are available.

## Abbreviations

YEOO: Yeast encapsulated orange oil
