# Supplementary figures and images for: Defining the mechanisms of action and mosquito larva midgut response to a yeast-encapsulated orange oil larvicide
Source: Parasit Vectors. 2022 May 28;15:183. doi: 10.1186/s13071-022-05307-6 (PMC9148471; doi:10.1186/s13071-022-05307-6)

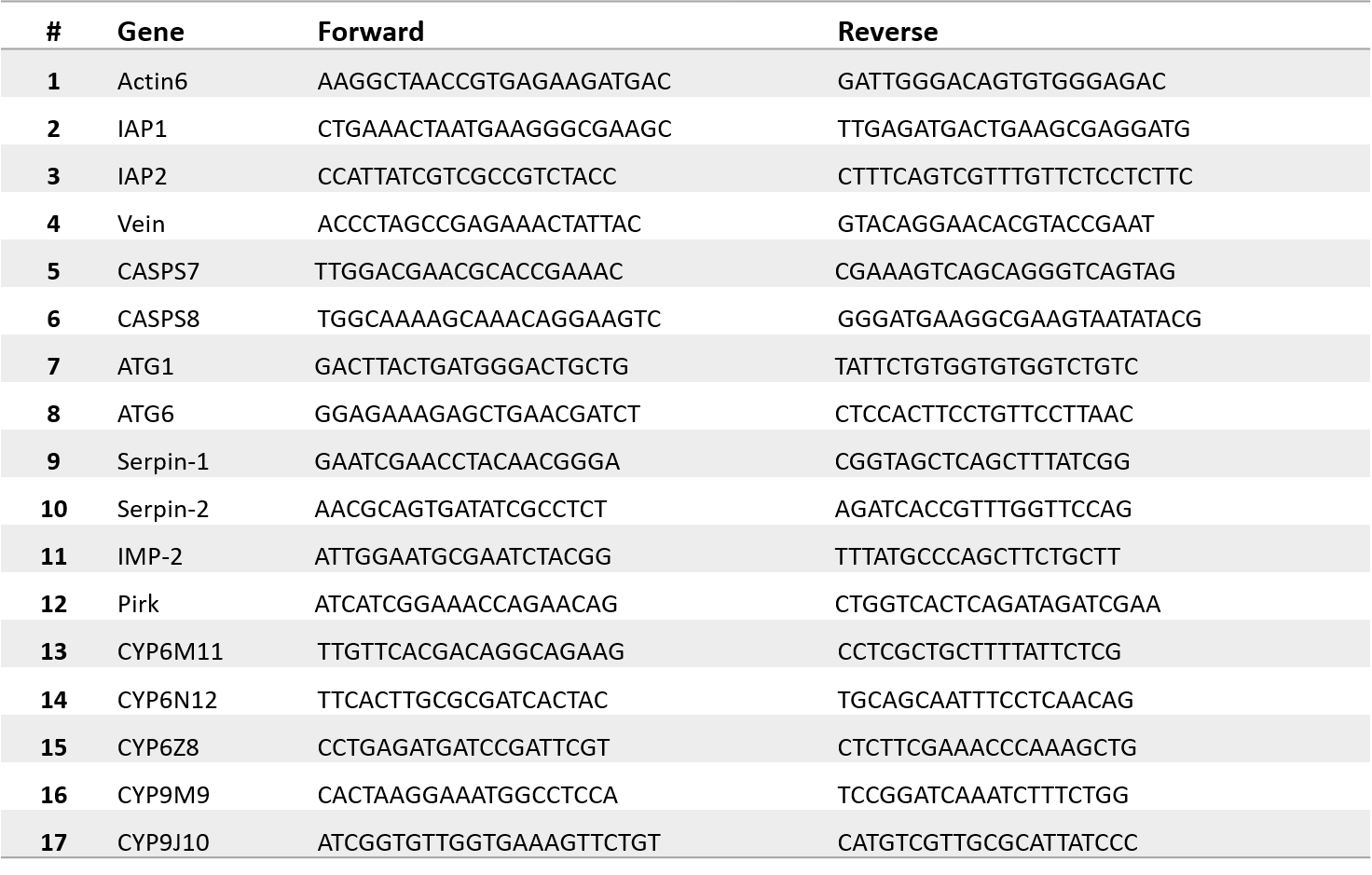

Supplement: Supplementary file 4 — Additional file 4: Table S1. Primer sequences used for amplification of target genes. [file 13071_2022_5307_MOESM4_ESM.tif]

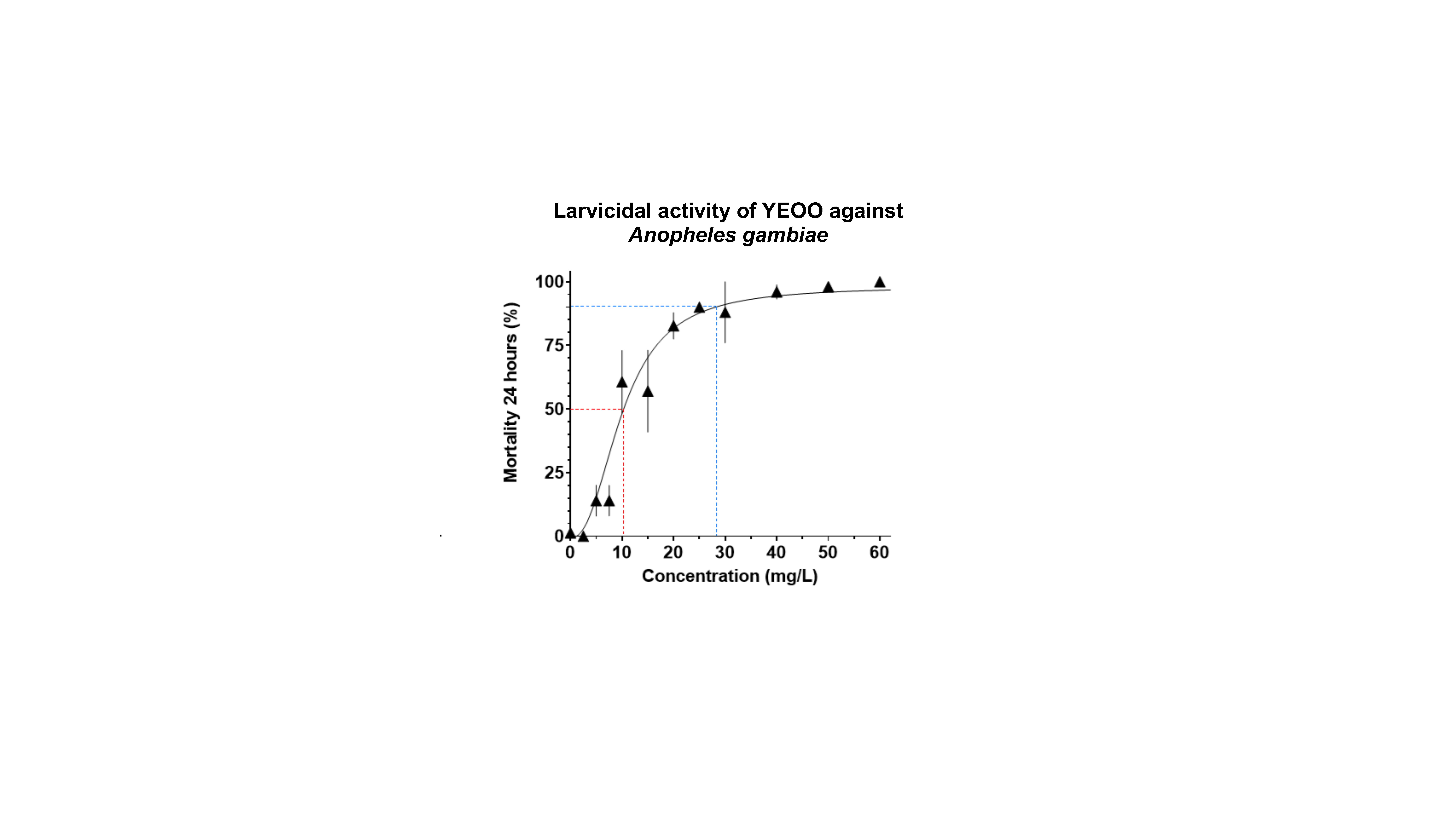

Supplement: Supplementary file 5 — Additional file 5: Figure S1. Bioassay of YEOO against Anopheles gambiae (G3), showing LC50 and LC90 of 10.3 mg/l and 28.1 mg/l respectively. [file 13071_2022_5307_MOESM5_ESM.png]
